# Selection on Horizontally Transferred and Duplicated Genes in *Sinorhizobium* (*Ensifer*), the Root-Nodule Symbionts of *Medicago*

**DOI:** 10.1093/gbe/evu090

**Published:** 2014-05-06

**Authors:** Brendan Epstein, Michael J. Sadowsky, Peter Tiffin

**Affiliations:** ^1^Department of Plant Biology, University of Minnesota; ^2^School of Biological Sciences, Washington State University; ^3^Department of Soil, Water, and Climate, University of Minnesota; ^4^BioTechnology Institute, Saint Paul, MN

**Keywords:** pangenome, mutation, purifying selection, fitness effects, structural variation

## Abstract

Structural variation, including variation in gene copy number and presence or absence of genes, is a widespread and important source of genomic variation. We used whole-genome DNA sequences from 48 strains of *Sinorhizobium* (recently renamed *Ensifer*), including 20 strains of *Sinorhizobium meliloti* and 12 strains of *S. medicae* that were the focus of the analyses, to study the fitness effects of new structural variants created by duplication and horizontal gene transfer. We find that derived duplicated and horizontally transferred (HT) genes segregate at lower frequency than synonymous and nonsynonymous nucleotide variants in *S. meliloti* and *S. medicae*. Furthermore, the relative frequencies of different types of variants are more similar in *S. medicae* than in *S. meliloti*, the species with the larger effective population size*.* These results are consistent with the hypothesis that most duplications and HT genes have deleterious effects. Diversity of duplications, as measured by segregating duplicated genes per gene, is greater than nucleotide diversity, consistent with a high rate of duplication. Our results suggest that the vast majority of structural variants found among closely related bacterial strains are short-lived and unlikely to be involved in species-wide adaptation.

## Introduction

The study of genomic variation within species has focused primarily on variation in nucleotide sequence. However, genomes also vary in structure (e.g., Zhang et al. 2009; Alkan et al. 2011; Sloan and Moran 2013) and gene content (e.g., Perna et al. 2001; Tettelin et al. 2005; Swanson-Wagner et al. 2010). In eukaryotes, there is substantial variation in gene copy number and gene presence (e.g., Emerson et al. 2008; Cridland and Thornton 2010; Swanson-Wagner et al. 2010), and in humans it is estimated that more nucleotide bases are affected by structural variants than by single-nucleotide polymorphisms (SNPs) (reviewed in Zhang et al. 2009; Alkan et al. 2011). Although gene content variation is common in eukaryotes, it is likely a more important source of genomic variation in bacteria, in which horizontal gene transfer (HGT) is common (reviewed in Gogarten and Townsend 2005; Kuo and Ochman 2009b; Zhaxybayeva and Doolittle 2011).

Horizontally transferred (HT) genes provide the genetic basis for some important bacterial adaptations, including symbiosis with eukaryotes (Sullivan and Ronson 1998; Ochman and Moran 2001; Blyton et al. 2013), antibiotic resistance (Ochman et al. 2000; Forsberg et al. 2012), and the ability to metabolize novel substrates (Pál et al. 2005). Despite a clear selective advantage of some HT genes, the majority of HT genes segregating in bacterial lineages are found at low frequency and have a short life span (Gogarten and Townsend 2005; Hao and Golding 2006; Achtman and Wagner 2008; Van Passel et al. 2008; Kuo and Ochman 2009b), consistent with strong purifying selection on gene content. Purifying selection may result from fitness costs due to transferred gene products interfering with protein interaction networks (Ochman et al. 2007; Wellner et al. 2007; Cohen et al. 2011), altered gene dosage (Sorek et al. 2007), misfolding of transferred gene products (reviewed in Baltrus 2013), transcription and translation of unnecessary genes (Stoebel et al. 2008), or transferred genes harboring deleterious mutations (reviewed in Moradigaravand and Engelstädter 2013). However, low frequencies and short residence times are not enough to conclude that HT genes are subject to purifying selection, because neutral mutations subject only to drift are also expected to be primarily rare and have short residence times in populations (Hartl and Clark 1997).

Similar to horizontal transfer, gene duplications can be an important source of genomic variation in prokaryotes (Hooper and Berg 2003; Andersson and Hughes 2009), and duplicated genes have been shown to confer adaptation to high temperatures (Riehle et al. 2005) and reduced nutrient concentrations (reviewed in Andersson and Hughes 2009) and the ability to metabolize novel substrates (Blount et al. 2012). Despite their adaptive potential, population genetic analysis (Emerson et al. 2008) and approaches based on the age distribution of duplicated genes within a single genome in both eukaryotes (Lynch and Conery 2000) and prokaryotes (Hooper and Berg 2003) indicate that most duplications are rapidly deleted or mutated into pseudogenes. Partial duplication also may disrupt coding sequences, causing frameshifts or null alleles with potentially large deleterious effects (Emerson et al. 2008).

Directly measuring the effect of mutations that occur in laboratory populations can be a powerful approach for examining the fitness consequences of individual mutations in specific environments. However, empirical manipulations are difficult to apply at a genomic scale, are usually conducted in unnatural environments, and do not provide insight into the actual history of mutations in natural populations. In contrast, the frequency of SNPs, duplications, and HT genes segregating in natural populations can be used to infer the relative importance of purifying and positive selection acting on genomic variation (reviewed in Eyre-Walker and Keightley 2007), even if this approach does not establish the specific biological functions of adaptive mutations or the biological costs responsible for purifying selection. These population genetic methods can be applied to genome-wide data to compare the evolutionary forces that have acted in natural populations on various types of mutations—unbiased with regard to choice of genes or experimental conditions.

To better understand the relative importance of positive and purifying selection acting on duplicated and HT genes, we analyzed whole-genome DNA sequence data from 20 strains of the alpha-Proteobacterium *Sinorhizobium* (recently renamed *Ensifer*) *meliloti*, 12 strains of *Sinorhizobium medicae*, and one or two strains from each of three other *Sinorhizobium* spp.: *S. fredii*, *S. saheli*, and *S. terangae*. All five species are nitrogen-fixing symbionts of legume plants, and *S. meliloti* and *S. medicae* are the most closely related within this sample (Sugawara et al. 2013; supplementary fig. S1, Supplementary Material online). Our specific objectives were to 1) characterize the extent of recent HGT and gene duplication in each species and 2) compare selection on new gene duplications and new HT genes to selection on nonsynonymous nucleotide variants. We also inferred the distribution of fitness effects (DFE) of nonsynonymous mutations as a point of comparison to evaluate the fitness effects of duplicated and HT genes.

## Materials and Methods

As described in Epstein et al. (2012) and Sugawara et al. (2013), we obtained full-genome sequence data from 48 strains of *Sinorhizobium*: 32 *S. meliloti* (which we filtered down to 20 strains—see Results and supplementary methods, Supplementary Material online) and 12 *S. medicae* strains that are the focus of this study, and two *S. fredii*, one *S. saheli*, and one *S. terangae* strains that are used as outgroups to identify HT genes. De novo genome assemblies were constructed from 90 bp Illumina reads using Abyss (Simpson et al. 2009), which yielded 131–528 contigs per strain and an average coverage of 140× (supplementary table S1, Supplementary Material online). The genomes were annotated by using MaGe (Vallenet et al. 2009). The annotations and assemblies are available at www.genoscope.cns.fr/agc/microscope/ SinorhizoScope (last accessed June 15, 2012) and in Genbank under BioProject PRJNA172127, and the reads are available from NCBI SRA048718. These data and assemblies have already been presented in Epstein et al. (2012) to examine the nucleotide diversity of the core genome and Sugawara et al. (2013) to study the functional gene content diversity.

Below, we describe how we identified duplicated genes, HT genes, and synonymous and nonsynonymous SNPs and then describe statistical analyses applied to each type of variant. We looked for derived variants in *S. meliloti* and *S. medicae*—those that likely arose since the most recent common ancestor of *S. meliloti* and *S. medicae*—to construct allele frequency spectra. Because *S. meliloti* shows signs of strong population structure in parts of the genome (Epstein et al. 2012), we conducted our analysis with a 24 strain subpopulation, which we filtered down to 20 strains (see Results and supplementary methods, Supplementary Material online) after identifying variants in the full set of strains.

### Identification of Duplicated Genes

The mutational processes that generate duplications in bacteria usually produce tandem copies (reviewed in Romero and Palacios 1997). De novo assembly often collapses tandem repeats and can also erroneously expand single-copy sequence (Salzberg et al. 2011). Consequently, we identified copy-number variation by aligning reads to a reference genome and searching for regions with significantly greater than average coverage. Both the *S. meliloti* reference genome (strain 1021; Galibert et al. 2001) and the *S. medicae* reference genome (strain WSM419; Reeve et al. 2010) have three large replicons: A chromosome and two large plasmids, which are referred to as pSymA and pSymB, following the notation of Galibert et al. (2001). *Sinorhizobium medicae* WSM419 also has a smaller plasmid (pSMED03) that we did not include in the tests for duplication because the population-wide coverage of this plasmid is very low (Epstein et al. 2012), presumably because this plasmid is absent from many of the *S. medicae* strains we sequenced. The reads from each strain were aligned to the reference genome of the same species using GSNAP (Wu and Nacu 2010) in paired-end mode, allowing up to eight mismatches per read and treating indel-openings as three mismatches, as described by Epstein et al. (2012). We used samtools rmdup (Li et al. 2009) to remove polymerase chain reaction duplicates, which removed 0.5–19% of reads (<2% of reads from the majority of strains). The depth of coverage across the genome was estimated for 100-bp windows by counting the number of aligned reads that started in each window. Reads that aligned equally well to multiple locations in the reference genome were counted fractionally (i.e., a read that was aligned to two locations was counted as half a read at both locations). Thus, repetitive regions of the reference genome were expected to have the same coverage as the rest of the genome if their copy number was the same as in the reference (Yoon et al. 2009).

We used the coverage-based method described by Yoon et al. (2009) to adjust coverage for GC content and identify 100-bp genomic windows with altered copy number relative to the reference genome (full details are in the supplementary methods, Supplementary Material online). After the initial identification of windows with altered copy number, contiguous stretches of duplicated windows with mean coverage <1.5, 1.8, or 2.0 times the mean coverage of the replicon were removed (the effects of using different filters are presented in the supplementary methods, Supplementary Material online). This filtering reduces the false-positive rate, but may increase the false-negative rate and also makes the approach insensitive to copy number differences in large gene families. Genes that had the majority of their sequence in a duplicated region were considered duplicated genes.

Duplications were considered “derived” if they affected genes that were present in both *S. meliloti* and *S. medicae* but were only duplicated in one of the species; these are likely duplications that occurred after the species split. For this purpose, orthology between genes in the *S. meliloti* and *S. medicae* reference genomes was inferred using the MaGE phyloprofile tool (www.genoscope.cns.fr/agc/miscroscope/compgenomics/phyloprofil.php, last accessed March 25, 2013): Syntenic genes that were ≥90% identical in amino acid sequence along >95% of gene length were considered orthologs. To identify fixed differences in gene copy number between *S. medicae* and *S. meliloti*, we identified gene families (defined as loci with >95% amino acid identity along >95% of gene length) within each reference genome. Gene families that differed in the number of copies between species, and for which coverage statistics revealed no evidence of copy number variation within species were considered fixed differences.

### Identification of HT Genes

We used the 34,150 gene clusters reported in Sugawara et al. (2013) to find HT genes. These clusters were identified by using CD-hit (Li and Godzik 2006) to cluster gene models annotated in the de novo assemblies of all 48 resequenced strains with 70% amino acid identity. Sequences within each cluster were then aligned to one another using muscle (Edgar 2004) and were used to identify HT genes and nucleotide variants, as described below. Gene clusters were assigned to replicons (chromosome, pSymA, pSymB, “ambiguous,” or “unmatched”) using MUMmer (Kurtz et al. 2004) (see supplementary methods, Supplementary Material online). In bacteria, “HGT” refers to both homologous recombination, which results in the exchange of orthologous genes between evolutionary lineages, and transfer of a gene from one evolutionary lineage to another. In this work, we focus on only the second type of HGT—that is, genes that were acquired by either *S. meliloti* or *S. medicae* from other evolutionary lineages and not from their most recent common ancestor. Because *S. meliloti* and *S. medicae* are the most closely related taxa in our sample (supplementary fig. S1, Supplementary Material online), any gene acquired after their split should be missing from the other three species. Thus, we identified HT genes as genes that were present in only *S. medicae* or only *S. meliloti*. This does not identify every gene that may have been involved in HGT at some point in its evolutionary history, but we are interested in derived HT genes—those acquired after the *meliloti–medicae* split.

### Identification of Nucleotide Variants

Several filters were applied to the gene clusters before identifying synonymous and nonsynonymous variants. First, we retained genes that were present in the core genome of the ingroup and at least one strain in the outgroup (using *S. meliloti* as the outgroup for *S. medicae* and *S. medicae* as the outgroup for *S. meliloti*). Second, to make data processing easier, we removed genes that were present in more than one copy in the de novo assemblies. As mentioned above, copy number in de novo assemblies is unreliable, so many of the genes were likely not really duplicated. However, the diversity statistics and frequency spectra obtained here are very similar to frequency spectra based on less stringent filtering criteria (Epstein et al. 2012). Third, we removed 16 genes that were likely involved in recombination between *S. meliloti* and *S. medicae* using two tests: 1) The ratio of fixed differences:shared polymorphisms was greater than 0.2 (following Epstein et al. 2012) or 2) the sequence nearest neighbor test statistic (Hudson 2000) was less than the maximum possible value. Fourth, we removed an additional 37 *S. meliloti* and 59 *S. medicae* genes that may have been involved in HGT with distantly related lineages by removing genes that were in the top percentile of synonymous divergence between *S. meliloti* and *S. medicae* (divergence was calculated using libsequence [Thornton 2003]) (Treangen and Rocha 2011). Finally, to ensure that we had high-quality alignments, we split any gene clusters into groups with at least 70% identity along at least 70% of the length of the gene (the CD-hit clustering did not include a length filter), and then removed any genes in which more than 10% of the positions had gaps (247 *S. meliloti* and 201 *S. medicae* genes).

### Diversity of Duplicates and Nucleotide Variants

In order to compare population-level diversity of duplicates to diversity of nucleotide sequences, we modified two standard estimates of nucleotide diversity: *θ*_w_, the average number of segregating sites (Watterson 1975), and *θ*_π_, the average pairwise nucleotide diversity (Tajima 1989) for duplication events (hereafter referred to as *θ*_w dup_ and *θ*_π dup_) by using segregating duplicates instead of segregating sites and treating the number of genes in the reference genome as the number of sites sampled. We also used estimates of *θ*_w dup_ and *θ*_π dup_ to calculate a modified (i.e., duplicate-based) Tajima’s *D* (*D*_T dup_) statistic. For comparison, we also estimated *θ*_w_, *θ*_π_, and *D*_T_ for biallelic 4-fold synonymous (*θ*_w syn_, *θ*_π syn_, and *D*_T syn_) and nonsynonymous (*θ*_w non__syn_, *θ*_π non__syn_, and *D*_T non__syn_) nucleotide sites.

### Construction of Frequency Spectra

We created a derived allele frequency spectrum for *S. meliloti* duplicates using *S. medicae* as the outgroup, and *S. medicae* duplicates using *S. meliloti* as the outgroup. To construct the derived duplication frequency spectra, we used only genes with a single ortholog and for which the ortholog showed no evidence of duplication in the outgroup population (28 out of 317 *S. meliloti* and 258 *S. medicae* duplicates were segregating in both species). Because we identified duplications relative to a reference genome, the allele frequency spectrum is biased toward low frequency variants. To correct for this bias, each frequency bin (the proportion of strains in which the duplication was detected) was divided by 1−*P*, where *P* is the probability that a duplication at frequency *P* is undetectable because it is in the reference genome (Emerson et al. 2008; Schrider and Hahn 2010).

For the 1,736 *S. meliloti* and 1,683 *S. medicae* core genes that passed the filters described above (the number of genes differs because we required the gene to be present in all ingroup strains but not all outgroup strains), the allele frequency spectra for nonsynonymous and 4-fold synonymous sites were inferred using the first step of the DFE-adaptive pipeline (Schneider et al. 2011). Sites segregating more than two alleles or that were polymorphic within the outgroup (*S. medicae* as the outgroup for *S. meliloti* and *S. meliloti* as the outgroup for *S. medicae*) were removed.

To test whether the allele frequency spectra of HT and duplicated genes differed from nonsynonymous SNPs, we performed a permutation test on pairs of variant classes: Nonsynonymous versus HT genes, synonymous versus HT genes, nonsynonymous versus duplicated genes, and synonymous versus duplicated genes. For each permutation, we pooled variants from both classes, drew two random samples without replacement (each the same size as the original samples), and calculated the difference in frequencies for each allele frequency class. For each comparison, we conducted 1,000 permutations.

We estimated the proportion of nonsynonymous point mutations that are deleterious using the method described by Eyre-Walker et al. (2006), which uses the minor allele frequency spectrum to estimate the proportion of mutations that are effectively neutral, weakly, moderately, or strongly deleterious. This analysis was run with a minimum of 1 million iterations. We also inferred the DFE using the method described by Eyre-Walker and Keightley (2009), which uses divergence data in addition to segregating sites. For *S. meliloti*, the results from this method were qualitatively similar to results from the Eyre-Walker et al. (2006) method (not shown). For *S. medicae*, however, the chain did not converge even after 50 million iterations, and thus we obtained no results.

## Results

We used the depth of coverage of the reference genome to identify duplicated regions segregating within *S. medicae* and *S. meliloti*. We first found regions with significantly greater than average coverage, using the method described by Yoon et al. (2009). After exploring the effects of varying the stringency of the detection parameters, we removed four strains that were outliers for the number of duplications (see supplementary methods, Supplementary Material online, for details). We estimated that 0.4–1.4% of the genes found in individual strains are segregating duplicates. In total, 317 of 6,811 genes (5%) found in the *S. meliloti* reference genome (56 out of 3,520 chromosomal genes, 192 out of 1,597 pSymA genes, and 69 out of the 1,694 pSymB genes) and 258 of 6,758 genes in the *S. medicae* reference genome (93 out of 3,764 chromosomal genes, 144 out of 1,431 pSymA genes, and 21 out of 1,563 pSymB genes) were duplicated in at least one of the resequenced strains (table 1). Fewer than 15% of the duplicated genes were annotated as being transposon related. In *S. meliloti*, nucleotide diversity at both synonymous and nonsynonymous sites is approximately 3-fold greater on one half than the other half of the chromosome (table 1; Epstein et al. 2012), but this pattern was not seen for diversity of duplicates (table 1).

**Table 1:** Mean Percent of Genes Duplicated (among Strain Range Shown in Parentheses), Number of HT Genes, and Pairwise Diversity of Segregating Duplicates and Nucleotide Variants

	% Duplicates	HT Genes	*θ*_π dup_	*θ*_π non_	*θ*_π syn_	*D*_T dup_	*D*_T non_	*D*_T syn_
*Sinorhizobium meliloti*	0.6 (0.4–0.9)	10,247	0.010	0.0011	0.0079	−1.14	−0.97	−0.78
chr.-full	0.1 (0–0.5)	2,318	0.003	0.0006	0.0044	−1.80	−0.99	−0.87
chr.-1^a^	0.1 (0–0.6)	804^b^	0.002	0.0009	0.0073	–1.88	–0.79	–0.59
chr.-2^c^	0.2 (0–0.7)	1,014^b^	0.003	0.0003	0.0020	−1.64	−1.18	−1.23
pSymA	1.3 (0.6–2.6)	1,985	0.023	0.0017	0.0148	−1.30	−1.00	−0.46
pSymB	0.8 (0–1.4)	1,271	0.011	0.0022	0.0169	−0.12	−0.66	−0.33
*S. medicae*	0.8 (0.2–1.4)	4,521	0.012	0.0008	0.0038	−0.13	−0.28	−0.12
chr.	0.7 (0.1–1.6)	1,399	0.009	0.0007	0.0025	0.66	−0.06	0.15
pSymA	1.9 (0.4–3.8)	823	0.030	0.0019	0.0082	−0.46	−0.09	0.15
pSymB	0.2 (0.1–0.4)	515	0.003	0.0012	0.0070	−1.38	−0.71	−0.82

^a^Before position 1735000.

^b^Does not sum to 2,318 because some HT genes assigned to the chromosome had an ambiguous location.

^c^After position 1735000.

Among replicons, the proportion of genes duplicated was approximately 2-fold greater for pSymA (per strain average of 1.3% and 1.9% of genes in *S. meliloti* and *S. medicae*, respectively) than the chromosome (0.8% in *S. meliloti* and 0.2% in *S. medicae*) and approximately 2- to 5-fold greater than pSymB (1.5% and 0.5%). Interestingly, the genes showing no evidence of segregating duplicates had faster evolutionary rates (as estimated by *K*_a_ and *K*_s_) in both species and in all three replicons, although the difference was statistically significant only for certain replicons (table 2; supplementary fig. S2, Supplementary Material online). We found no duplications that were fixed differences between *S. meliloti* and *S. medicae*.

**Table 2:** Mean *K*_a_, *K*_s_, and *K*_a_/*K*_s_ between the *Sinorhizobium meliloti* and *S. medicae* Reference Genomes for Duplicated and Unduplicated Genes, Including Only Genes in Both *S. meliloti* and *S. medicae*

	Unduplicated				Duplicated		
	Count	*K*_a_	*K*_s_	*K*_a_/*K*_s_	Count	*K*_a_	*K*_s_
*S. meliloti*							
Chr.	1,963	0.027	0.40	0.068	17	0.020	0.35
pSymA	233	0.026	0.29	0.15	32	0.023	0.24
pSymB	666	0.032*	0.45	0.073	7	0.024*	0.40
*S. medicae*							
Chr.	1,961	0.027	0.40*	0.068	9	0.019	0.24*
pSymA	22	0.027*	0.32*	0.13	34	0.018*	0.17*
pSymB	690	0.031	0.44	0.075	2	0.018	0.26

*P < 0.05 (two-sided *t*-test for difference between duplicated and unduplicated genes).

Under the assumption that putative duplicates adjacent to one another may represent a common duplication event, we identified 212 duplications in *S. meliloti* and 143 in *S. medicae.* There was a mean of 14 events per *S. meliloti* strain and 23 events per *S. medicae* strain. The duplicated regions comprised a mean size of 2.1 genes in *S. meliloti* and 2.6 genes in *S. medicae*, with the largest of the duplicated regions containing 20 genes. In total, 80% and 81% of duplicated genes in *S. meliloti* and *S. medicae*, respectively, were found near another duplicated gene in at least one strain. Although frequency spectra constructed from these events were qualitatively similar to spectra constructed with genes (not shown), the population frequencies of genes within an event were not always the same, indicating that either these genes were not duplicated at the same time or that they may spread independently. For this reason, we only present results for frequency spectra constructed using genes.

### HT Genes

**Fig. 1.—:**
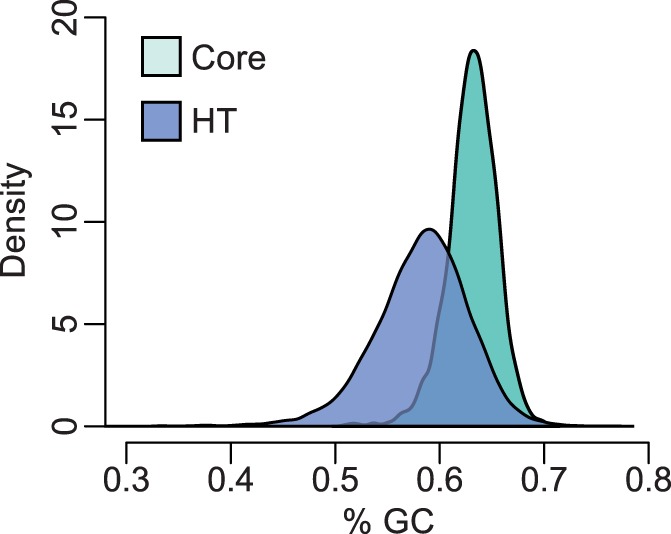
Distribution of GC content for HT genes and core genes. The distribution is shown for *Sinorhizobium meliloti* genes. *Sinorhizobium medicae* distributions are nearly identical.

Under the assumption that genes present in only one of the five species were recently acquired through HGT (HT), individual strains harbored 1,389–2,433 HT genes (∼20–35% of the genome), with a total of 10,247 HT genes in the 20 *S. meliloti* strains (2,318 chromosomal, 1,985 on pSymA, 1,271 on pSymB, 3,790 not matched to a reference replicon, and 883 ambiguously assigned) and 4,521 HT genes in the 12 strains of *S. medicae* (1,399 chromosomal, 823 on pSymA, 515 on pSymB, 1,586 unmatched, and 198 genes with an ambiguous assignment). As expected if these genes originated in distantly related taxa, the distribution of GC content for the putatively HT genes was very different from the distribution of GC content for the core genome (fig. 1). We found far more HT than duplicated genes that were fixed in one species and absent in the other; approximately 9% of HT genes were fixed in *S. medicae* (394 genes) and 3.3% fixed in *S. meliloti* (341 genes), comprising approximately 5–8% of the genome of each species.

**Fig. 2.—:**
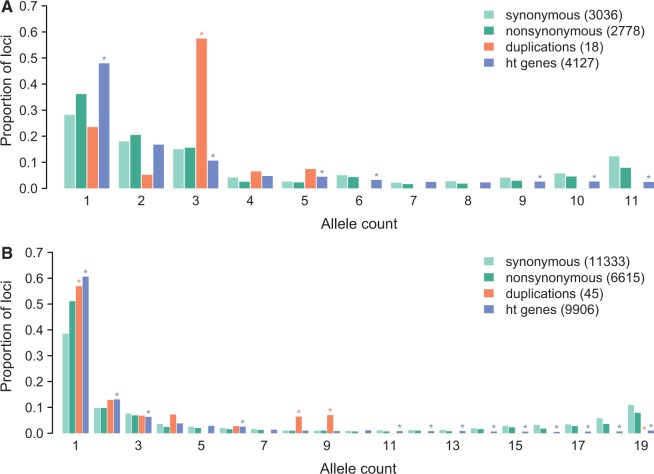
Derived allele frequency spectrum for four classes of mutations. (*A*) *Sinorhizobium medicae* and (*B*) *S. meliloti*. The *y* axis is the proportion of sites (for synonymous and nonsynonymous SNPs) or genes (for duplications and HGTs) within a class of mutations. Duplication and HT gene bars marked with an asterisk (*) are significantly different from synonymous sites. The values in the legends are the number of segregating sites for nucleotide variants or segregating genes for duplications and HT genes used to construct the spectra. Only derived duplications and nucleotide sites for which the ancestral state could be confidently inferred were used to construct the spectra.

There was significant variation in the fixation of HT genes among clusters of orthologous groups (COG) categories (χ^2^ contingency test df_meliloti_ = 16, df_medicae_ = 9, *P* < 0.001). Notably, genes involved in transport and metabolism were significantly over-represented among the fixed HT genes (fig. 2), whereas genes involved in DNA replication and repair were under-represented. Although genes assigned to COG categories related to transcription and translation had the greatest proportion of fixed HT genes, many of these genes were annotated as “unknown function,” suggesting that the COG classification was uncertain.

HT genes tended to cluster: More than half of HT genes were within three genes of another HT gene. The largest of the HT spatial clusters harbored 47 and 99 genes, with an average of 2.8 and 3.8 genes in *S. medicae* and *S. meliloti*, respectively. In both species, there was a highly significant negative correlation between the population frequency of a gene and the median size of the spatial cluster in which it was found (*S. meliloti*: *r*_df_ = 10,176 = −0.17, *S. medicae*: *r*_df_ = 4,481 = −0.15, both *P* < 0.0001).

### Excess of Low Frequency Duplications and HT Genes

**Fig. 3.—:**
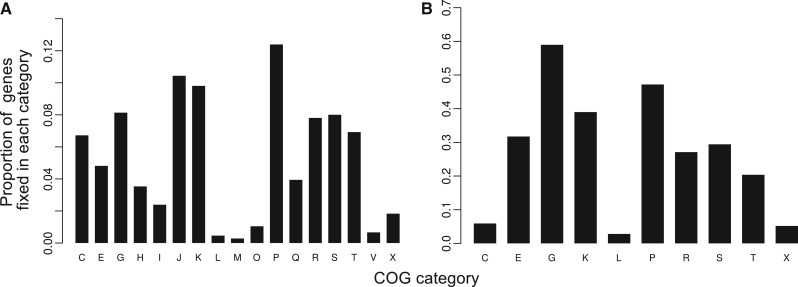
Proportion of HT genes in each COG category that are fixed. (*A*) *Sinorhizobium meliloti* and (*B*) *S. medicae*. Only categories with at least 50 HT genes were included.

As expected, nonsynonymous mutations in both *S. meliloti* and *S. medicae* showed an excess of low-frequency variants and a scarcity of high frequency variants, relative to synonymous mutations (fig. 3). This is consistent with stronger purifying selection acting against nonsynonymous mutations. There was approximately 2-fold greater synonymous and nonsynonymous site diversity (*θ*_w_ and *θ*_π_) found in *S. meliloti* than in *S. medicae*, indicating that *S. meliloti* had an approximately 2-fold larger effective population size (assuming *N*_e_ is directly proportional to *θ*; table 1). Based on the minor allele frequency spectrum, 10% of nonsynonymous mutations in *S. meliloti* are effectively neutral, whereas nearly two-thirds are subject to very strong (probably negative) selection (table 3). The *S. medicae* strains harbored a larger portion of putatively neutral and weakly deleterious mutations, consistent with a negative relationship between the efficacy of selection and effective population size. The proportion of strongly deleterious mutations, however, was similar in the two species.

**Table 3:** DFE of Nonsynonymous Mutations: Percent of Sites in Each Selection Bin (Standard Error)

	*N*_e_*s*			
Species	0–1	1–10	10–100	>100
*Sinorhizobium meliloti*	10 (0.4)	8.3 (0.4)	15 (1.2)	66 (1.4)
*S. medicae*	19 (0.7)	6.7 (0.8)	9 (1.5)	65 (2)

For all three replicons in *S. meliloti*, *D*_T__dup_ was more negative than *D*_T non__syn_ (table 1), and segregating derived duplications were found almost entirely at low frequency (fig. 3). The pattern is not as clear in *S. medicae*, in which values of *D*_T dup_, *D*_T non__syn_, and *D*_T__syn_ were similar to one another, consistent with less effective purifying selection in this species with a smaller effective population size. Likewise, in both species segregating derived duplications were found at lower frequency than segregating synonymous polymorphisms in all replicons, although the differences were not significant in *S. meliloti* pSymB or the *S. medicae* chromosome. Segregating derived duplications were also found at lower frequency than nonsynonymous polymorphisms in most replicons (the exceptions were *S. meliloti* pSymB and the *S. medicae* chromosome) and genome-wide (table 4 and fig. 3), but these differences between nonsynonymous sites and duplications were statistically significant only in *S. meliloti* (table 4). Interestingly this pattern did not hold for duplications of genes found in only one of the reference genomes; for these genes, there was an excess of intermediate frequency variants (supplementary fig. S3, Supplementary Material online), although there were still very few at high frequency.

**Table 4:** Frequencies of Segregating Synonymous (S) and Nonsynonymous (NS) SNPs, HT Genes (HT), and Duplicated Genes (Dup) Segregating in *Sinorhizobium meliloti* and *S. medicae* and Results of Randomization Tests to Determine Whether Duplications and HT Genes Are Segregating at Lower Mean Frequency than S and NS SNPs (Significant Values Are in Bold)

	S	NS	Dup	HT	NS versus Dup^a^	NS versus HT^a^	S versus Dup^a^	S versus HT^a^
*S. meliloti*	0.34	0.26	0.13	0.14	**0.002**^b^	**0**^b^	**0**^b^	**0**^b^
Chr.	0.37	0.28	0.06	0.18	**0.01**^b^	**0**^b^	**0**^b^	**0**^b^
pSymA	0.25	0.22	0.13	0.20	**0.05**	0.09	**0.01**	**0**
pSymB	0.32	0.25	0.27	0.19	0.63	**0**	0.05	**0**
*S. medicae*	0.36	0.30	0.22	0.24	0.15	**0**	**0.03**	**0**
Chr.	0.30	0.24	0.29	0.29	0.85	1.0	0.76	0.21
pSymA	0.36	0.32	0.21	0.32	0.77	0.75	**0.01**	0.05
pSymB	0.42	0.38	—^c^	0.30	—	**0**	—	**0**

Note.—Duplicated genes were included only if they were present as a single copy in one species and duplicated in the other.

^a^Randomization tests were performed by randomly assigning the total number of variants to a specific variant class (e.g., NS or Dup) and then comparing the difference in the number of counts in each class to the actual difference; values shown are the proportion of 1,000 randomizations in which the difference in frequency of NS (or S) SNPs compared with duplications (or HT) genes was greater than the true difference.

^b^Indicates that the relative difference between the mean frequency of HT genes or duplications and nucleotide variants was significantly (*P* < 0.05) greater in *S. meliloti* than in *S. medicae*. The point estimates of the relative differences were greater in *S. meliloti* for all comparisons except duplications on pSymA.

^c^No derived duplications on pSymB.

Similar to the patterns found for duplicated genes, in both species there was a significantly greater proportion of singleton HT genes than synonymous SNPs and fewer common HT genes than synonymous mutations, genome-wide (fig. 3). These patterns are largely consistent across replicons (supplementary fig. S4, Supplementary Material online). In both species and all replicons, the mean segregating frequency of HT genes was lower than synonymous SNPs, although the differences are not significant for the *S. medicae* chromosome or *S. medicae* pSymA (table 4). In addition, the mean segregating frequency of HT genes is lower than nonsynonymous SNPs in all of *S. meliloti*, although this difference is not significant in pSymA (table 4). Approximately one-third of HT genes could not be assigned to a replicon; these unassigned genes also segregated at very low frequency.

## Discussion

Whole-genome sequencing of multiple strains from two *Sinorhizobium* species, *S. meliloti* and *S. medicae*, was used to investigate the extent of HGT and gene duplication and to compare the strength of selection acting on new HT and duplicated genes to selection acting on new nucleotide mutations. By comparing the derived allele frequency spectra, we found that both gene duplications and HT genes showed an excess of low frequency variants and lower mean frequency relative to synonymous SNPs across the genome, and nonsynonymous SNPs in parts of the genome. These results suggest that there is strong purifying selection acting against HT and duplicated genes. We also found that the apparent strength of purifying selection was stronger in *S. meliloti* than *S. medicae*, consistent with the apparently larger effective population size of *S. meliloti*.

As a baseline by which to compare the relative strengths of purifying selection acting on duplicated and HT genes, we first inferred the DFE of nonsynonymous substitutions and found that about two-thirds are under strong purifying selection and 10% (*S. meliloti*) to 20% (*S. medicae*) are effectively neutral. In *Escherichia coli* and *Salmonella enterica*, Charlesworth and Eyre-Walker (2006) estimated that at only approximately 3% of nonsynonymous mutations were effectively neutral (also reviewed in Eyre-Walker and Keightley 2007). Charlesworth and Eyre-Walker (2006) found that segregating nucleotide diversity, as measured by *θ*_w_, was approximately an order of magnitude greater in these species than in the species we studied here. The differences in the efficacy of purifying selection are consistent with differences in effective population sizes between these species and also underscore that selection may not be highly effective in all prokaryotic species.

### HT Genes

We found that most species-specific genes in *S. meliloti* or *S. medicae* were segregating at low frequency. Because these genes are found in only one out of the five closely related *Sinorhizobium* species we examined, and because they have a different GC content distribution than core genes (fig. 1), these genes were likely gained by HGT. It is possible that some of these genes have been gained by de novo gene evolution, but de novo origination of genes is thought to be quite rare (Wu et al. 2011), whereas HGT is thought to be quite common (e.g., Gogarten and Townsend 2005). Many previous comparative genomics studies have found that most genes in the noncore portion of a bacterial pangenome are rare (reviewed in Gogarten and Townsend 2005; Touchon et al. 2009). Studies that examined the fate of HT genes by mapping gain and loss onto a phylogeny (Hao and Golding 2006; Van Passel et al. 2008), by the age distribution of HT genes inferred from codon usage data (Lawrence and Ochman 1998), by fitting models of gene gain and loss to gene presence data (Collins and Higgs 2012; Lobkovsky et al. 2013), and by the age distribution of pseudogenes (Liu et al. 2004; Kuo and Ochman 2010) suggested that most genes acquired by HGT are rapidly lost, consistent with our finding that HT genes segregate at lower frequency relative to synonymous SNPs in all replicons in both species (fig. 2 and table 4; supplementary fig. S4, Supplementary Material online). To the extent that synonymous sites are selectively neutral, the skew toward rare variants is consistent with HT genes being primarily subject to purifying selection. In fact, the mean segregating frequency of HT genes is less than that of nonsynonymous SNPs in all *S. meliloti* replicons. Although demographic events and population structure can mimic the effects of selection, these factors will also affect the frequency spectra of nucleotide sites that we are using as a basis of comparison.

There are several other caveats to the interpretation of the excess of low frequency variants as an indication of purifying selection. One caveat is that bacterial genomes experience a higher neutral deletion rate than neutral insertion rate: Pseudogenes tend to accumulate more small deletions than insertions (Kuo and Ochman 2009a), and mutation accumulation experiments show high genome-wide deletion rates (Nilsson et al. 2005). Even in the absence of selection, this deletion bias could result in a skew toward rare HT and gene duplication variants if the deletion rate of neutral HT genes and duplications was greater than the back mutation rate of neutral nucleotide mutations. Because we conducted our analysis on two closely related species that differ in effective population size, we have the opportunity to distinguish between purifying selection and mutational bias.

Across the genome as a whole the frequency distribution of both HT and duplicated genes is more similar to the frequency distribution of nucleotide variants in *S. medicae* than in *S. meliloti* (fig. 3 and table 4), consistent with less effective purifying selection in *S. medicae*, the species with the smaller effective population size. To ensure that the differences between *S. medicae* and *S. meliloti* were not due to the difference in sample size, we randomly subsampled 12 strains from *S. meliloti* 100 times; the differences between the mean segregating frequency of nucleotide variants and structural variants were greater in at least 95% of the *S. meliloti* subsamples than in *S. medicae* in the genome as a whole and in the chromosome individually (table 4). This pattern is expected if purifying selection is causing the excess of rare HT genes and duplications. However, among the megaplasmids, the differences in mean frequency were not significantly (*P* < 0.05) greater in *S. meliloti* than in *S. medicae*. This is consistent with previous suggestions that purifying selection on the megaplasmids is weaker than on the chromosome (Epstein et al. 2012), possibly due to differences in gene expression levels (Morrow and Cooper 2012).

A second caveat is that many HT genes that are currently segregating could be beneficial, but only at a very local scale, so they remain at low frequency in the species as a whole (Hao and Golding 2006; Doolittle and Zhaxybayeva 2009)—the approach we used cannot distinguish between universally deleterious mutations and mutations that are locally beneficial, but deleterious on a larger scale. Nevertheless, our results clearly suggest that very few transferred genes offer widespread, long-term benefits, and the highly significant differences in the probability of fixation of HT genes among COG categories (fig. 2) indicate that HT genes are not fixed at random. For example, HT DNA replication and repair genes (COG category L) are very rarely fixed, whereas carbohydrate metabolism genes (COG category G) are frequently fixed. Recent work in other systems also suggests selection acting against new genes: There is an excess of young pseudogenes in natural populations of *Salmonella* (Kuo and Ochman 2010), and many deletions increase fitness in experimental populations of *Salmonella* (Koskiniemi et al. 2012) and *Methylobacterium* (Lee and Marx 2012). Taken together, the evidence that gene content evolves either neutrally or is subject to purifying selection suggests that few of the noncore genes found in bacterial pan-genomes are likely to confer fitness benefits across an entire species or contribute to differences among species.

### Duplicated Genes

We found that gene duplication polymorphism was greater than nucleotide diversity in *S. medicae*; population-level estimates of the number of gene duplicates per gene (*θ_π_*_ dup_) were 2- to 3-fold greater than the number of segregating sites per synonymous site (*θ_π_*_ syn_) in all replicons except pSymB (table 1). In contrast, in *S. meliloti*, the level of gene duplication diversity was similar to the level of nucleotide diversity (table 1), in spite of potentially strong purifying selection acting against new duplications. Even if only 70–80% of our duplications are real (see supplementary table S2, Supplementary Material online), and there are no duplications that we failed to identify, the genome-wide level of duplication diversity would be greater than (*S. medicae*) and similar to (*S. meliloti*) the level of nucleotide diversity. These results suggest that the rate of duplication is greater than the rate of base substitution, consistent with results from mutation accumulation experiments in *Caenorhabditis elegans* (Lipinski et al. 2011), the age distribution of paralogs in *E. coli* (Hooper and Berg 2003), and observations of spontaneous duplications in *Salmonella typhimurium* (Pettersson et al. 2009). A higher rate of duplications than point mutations may also explain why duplication diversity does not differ between the two halves of the *S. meliloti* chromosome—in contrast with the much greater nucleotide diversity in the first (position 1–1735000) than second half (Epstein et al. 2012; table 1). The sharp difference in nucleotide diversity was interpreted as possible evidence for a strong selective sweep—if that interpretation was correct, then the different patterns at nucleotide sites than duplications may be due to the higher mutation rate of duplications having already erased the evidence for the selective sweep.

Like HT genes, there are many duplications segregating within species, yet derived duplications tend to segregate at a lower frequency than synonymous SNPs. The short life span of most gene duplications has been noted previously. For example, Hooper and Berg (2003) looked at the age distribution of paralogs and concluded that most new duplications are rapidly deleted and estimated that only approximately 1/1,000 duplications are beneficial in *E. coli*. Laboratory studies of duplications in bacteria suggested that many reduce fitness (Pettersson et al. 2009; Reams et al. 2010), and results of a mutation accumulation experiment in *Drosophila melanogaster* indicated that >99% of duplications are deleterious (Schrider et al. 2013). The low segregating frequency of duplications that we found here suggests that duplications are mostly deleterious in *Sinorhizobium* as well, although this is subject to the same caveats (mutational bias, local adaptation, and variation among replicons) that apply to the HT genes.

Interestingly, duplications of genes found in only one of the two reference genomes were often found at intermediate frequency (supplementary fig. S3, Supplementary Material online). It is possible that these genes were gained by HT and then duplicated. If this is true, then copy number in HT genes may be under less constraint than in core genes. Alternatively, they could be genes that were duplicated in the ancestral genome, but then lost from the reference strain of one species. In addition, our finding that there are no recently fixed gene duplications is consistent with previous indications that HGT contributes more than duplication to differences among bacterial species in gene family size (Treangen and Rocha 2011).

### Conclusions

Despite the clear adaptive potential of some HT and duplicated genes, purifying selection appears to be the prevailing force acting against both duplications and HT genes in *Sinorhizobium*, at the level of the entire species. The prevalence of purifying selection in these bacteria is similar to the apparently strong purifying selection acting against the majority of duplications and structural variants in model eukaryotes (Emerson et al. 2008; Cridland and Thornton 2010; Li et al. 2011). If the prevalence of purifying selection in *Sinorhizobium* is representative of selection acting on HT and duplicated genes in other bacterial species then the majority of the differences between bacterial core and pan-genomes may not be adaptively important, but rather reflect new mutations that are unlikely to shape the long-term evolutionary trajectory of a species.

## Supplementary Material

Supplementary methods, figures S1–S5, and tables S1–S5 are available at *Genome Biology and Evolution* online (http://www.gbe.oxfordjournals.org/).

Supplementary Data
