# Supplementary material for: Selection on Horizontally Transferred and Duplicated Genes in Sinorhizobium (Ensifer), the Root-Nodule Symbionts of Medicago
Source: Genome Biol Evol. 2014 May 6;6(5):1199–209. doi: 10.1093/gbe/evu090 (PMC4040998; doi:10.1093/gbe/evu090)
Supplement: Supplementary Data [file supp_6_5_1199__index.html]

Selection on horizontally transferred and duplicated genes in Sinorhizobium (Ensifer), the root-nodule symbionts of Medicago — Selection on Horizontally Transferred and Duplicated Genes in Sinorhizobium (Ensifer), the Root-Nodule Symbionts of Medicago — Supplementary Data 

# Selection on Horizontally Transferred and Duplicated Genes in *Sinorhizobium* (*Ensifer*), the Root-Nodule Symbionts of *Medicago*

## Supplementary Data

files

**Files in this Data Supplement:**

- Supplementary Data - pdf file
