# Supplementary material for: Selection on Horizontally Transferred and Duplicated Genes in Sinorhizobium (Ensifer), the Root-Nodule Symbionts of Medicago
Source: Genome Biol Evol. 2014 May 6;6(5):1199–209. doi: 10.1093/gbe/evu090 (PMC4040998; doi:10.1093/gbe/evu090)
Supplement: Supplementary Data [file supp_evu090_Epstein_etal_Supplemental_Info_printed.pdf]

Supplementary Methods

Details of duplication identification

Genomic regions with especially high or low GC content tend to have lower coverage (Bentley et al. 2008), so we first corrected for this bias using the method described by Yoon et al. (2009). In particular, each of the windows in each replicon were binned by % GC content (e.g. windows with 1% GC, 2% GC, etc.), and then an adjustment factor for each GC bin ($adjustment\_factor[GC_i]$) was calculated as:

$$adjustment\_factor[GC_i] = M / [m(GC_{i\ +/-\ 3\%})] \quad (1)$$

where $[GC_i]$ is the i percent GC in the window, M is the median replicon-wide coverage and $m(GC_{i\ +/-\ 3\%})$ is the median coverage of windows with % GC content equal to i-3, i-2, i-1,…., i+3. For example, for windows with i = 50% GC, m would be equal to the median GC content of windows with 47 - 53% GC. The adjustment factors were first calculated separately for each strain (because average coverage varies among strains), then the mean adjustment factor for each GC bin, across strains, was used to calculate corrected coverage =

$$mean\_adjustment\_factor[GC_i] * coverage[GC_i]. \quad (2)$$

The corrected coverage was used to identify duplicated regions in each strain using the event-wise testing framework described by Yoon et al. (2009). In brief, for each strain, a Z-score was calculated for each window by subtracting the mean depth of coverage of the replicon and

dividing by the standard deviation of the depth of coverage. Because much of the reference *S. meliloti* pSymA is not found in the re-sequenced strains, we calculated the mean and standard deviation (separately for each strain) using only windows that had > 0 coverage in the strain being tested. This reduced the number of false positive duplications because the mean was not decreased by reference-strain-specific regions. The Z-score for each window in each strain was then converted to an upper-tail p-value, assuming a normal distribution, using the pnorm function in R (R Development Core Team 2013). Contiguous intervals of windows were considered to represent a single duplication event if:

$$max(p) < (FPR / (L / n))^{(1/n)} \quad (3)$$

where max(p) is the largest p-value of the windows in the interval, L is the number of windows on the replicon, n is the number of windows in the interval, and the false positive rate (FPR) = 0.001. We tested even-sized intervals from 2-16 (2, 4, 6, ..., 16), and intervals were slid along the replicons in steps equal to half the size of the interval. Finally, contiguous stretches of duplicated windows separated by one unduplicated window were combined into a single region. Genes that had the majority of their sequence in a duplicated region were considered duplicated genes.

To identify regions with reduced copy number relative to the reference genome we used the same approach we used to identify duplicated genes except the lower-tail p-value from the normal distribution was used when calculating the Z-score, and we filtered out regions that had more than 0.65, 0.55, or 0.5 times the mean coverage.

Assessment of error rates in duplication calls

We explored the accuracy of the duplication calls and the effect of varying the filtering

parameters in several ways. First, we re-sequenced the strain used to construct the *S. meliloti* reference genome, strain Rm1021, and ran it through the duplication pipeline. The reference genome was well captured by our sequencing methods: all sites were covered by reads and a *de novo* assembly recovered > 99.9% of the genome. We found that as the stringency of the filtering increased, the number of duplicated windows in the re-sequenced Rm1021 and the other re-sequenced strains decreased. The greatest decrease was between the 1.5X and 1.8X cutoffs (supplementary Table S3).

Second, we used additional sequence data from a collection of experimentally evolved strains derived from *S. meliloti* strains KH46c, Rm41, and USDA1002 (Epstein 2013; reads available from NCBI BioProject PRJNA233494). These evolved strains were separated from their progenitors by fewer than 200 generations, and therefore we would expect that most of the duplications present in the progenitors would be present in the evolved strains as well. Depending on parameter values, 55 - 79% of the duplications were identified in all or all but one of the descendant lines derived from KH46c and Rm41 (supplementary Table S2). The validation rate for strain USDA1002 was much lower, but this strain was subsequently removed due to an anomalously high number of duplications (see below). The 1.8X coverage showed the greatest consistency between progenitors and evolved lines. Third, we looked for "heterozygous" base calls in the duplicated regions, because if the copies of the duplicated regions have diverged in sequence, then we would expect to find sites in the duplicated regions with about half the reads supporting one base call and half supporting another (assuming two copies). On average, the proportion of "heterozygous" sites / total sites with the 1.8X cutoff was 27 (*S. meliloti*) – 53 (*S. medicae*) times greater in duplicated than in unduplicated regions (supplementary Tables S4 and S5). Based on these criteria, we chose 1.8X as the filter cutoff.

Based on the 1.8X coverage for calling duplicated regions, the re-sequenced reference strain has fewer duplications (relative to the original reference) than did any other strain (supplementary Table S3, and there were four *S. meliloti* strains that were clearly outliers (Supplementary Figure S5). The outlier strains included USDA1002, which had the lowest validation rate based on the evolved lines, as well as strains HM006-1, M162, and T073. For the rest of the results, we excluded these four outlier strains.

Assignment of genes to replicons

Based on nucleotide diversity, the chromosome and two large plasmids of each species appear to have different evolutionary histories - nucleotide diversity is 2- to 3-fold greater on the megaplasmids than the chromosome, possibly due to relaxed selective constraint on plasmid genes (Epstein et al. 2012). For this reason, we attempted to assign genes from the *de novo* assemblies to a reference replicon. There were two stages: first, we mapped contigs from the *de novo* assemblies to the reference genome using MUMmer (Kurtz et al. 2004). Then we assigned gene clusters to replicons based on the assignment of the contigs on which the genes were located. Contigs were assigned to a replicon if the contig had ≥ 80 % identity to the reference genome along ≥ 20% of the contig length. If these criteria were not met, the contig was considered unmatched. Unmatched contigs may be from small accessory plasmids not present in the reference genome or from regions with large numbers of HT genes not present in the reference. Contigs that matched multiple replicons were assigned to the single best match if the best match was at least twice the length of the other matches combined, otherwise the contig assignment was considered ambiguous. Then gene clusters were assigned to a particular replicon if ≥ 20% of the cluster members were on a contig assigned to the same replicon, and no more

than one third of the cluster members with a replicon assignment were assigned to a different replicon. If more than one third of the cluster members were on a contig assigned to a different replicon, the gene cluster was considered "ambiguous."

Construction of the phylogeny

As described in Sugawara et al. (2013), we constructed a phylogeny for all 48 re-sequenced strains, and the reference genomes using 645 protein-coding genes found in all *Sinorhizobium* strains and *R. leguminosarum* WSM1325. Phylip (Felsenstein 1989) was used to calculate evolutionary distances using the F84 model and assemble a neighbor-joining tree. We mapped the support values from 1000 bootstrapped datasets on to the tree created from the whole dataset using the sumtrees program (Sukumaran and Holder 2010), and collapsed splits with < 60% support to polytomies.

Supplementary Tables and Figures

Table S1. Number of contigs, *de novo* genome assembly size, and number of predicted protein coding genes for all 48 *Sinorhizobium* (*Ensifer*) strains. This information is also available from Sugawara et al. (2013). *S. meliloti* and *S. medicae* strains used to constuct the frequency spectrums are bolded. Note that duplicated genes were identified by aligning reads to the reference genome, while the rest of the statistics refer to the *de novo* assembly. The first 24 *S. meliloti* strains (through USDA1021) are the focal sub-population.

Strain	Contigs	Assembly size (Mb)	Median contig size	Total number of CDS	Number of HT CDS	Number of duplicated genes
S. meliloti						
HM006-1	528	7.04	9,209	7,560	1,686	105
**HM007-12**	**156**	**7.27**	**18,030**	**7,775**	**1,807**	**36**
**HM007-17**	**131**	**7.30**	**26,383**	**7,710**	**1,838**	**35**
**HM013-1**	**168**	**6.98**	**24,663**	**7,384**	**1,763**	**38**
**HM015-1**	**202**	**7.23**	**16,627**	**7,662**	**1,911**	**51**
**KH16b**	**242**	**7.01**	**19,570**	**7,482**	**1,737**	**42**
**KH46b**	**171**	**6.88**	**25,269**	**7,255**	**1,777**	**24**
**KH46c**	**251**	**7.08**	**18,053**	**7,644**	**1,843**	**35**
**M156**	**229**	**7.02**	**16,410**	**7,480**	**1,929**	**38**
M162	234	7.20	13,996	7,784	1,579	107
**M195**	**162**	**7.18**	**25,400**	**7,617**	**1,848**	**26**
**M210**	**190**	**7.15**	**21,611**	**7,574**	**1,833**	**32**
**M243**	**154**	**7.13**	**27,153**	**7,509**	**1,703**	**35**
**M249**	**273**	**7.23**	**7,227**	**7,849**	**1,972**	**34**
**M268**	**339**	**7.27**	**12,089**	**7,833**	**2,025**	**58**
**M270**	**377**	**7.84**	**6,342**	**8,858**	**2,433**	**64**
**M30**	**227**	**7.19**	**15,309**	**7,734**	**1,867**	**24**
**N6B1**	**297**	**7.60**	**10,829**	**8,277**	**1,930**	**56**
**N6B7**	**275**	**7.70**	**14,564**	**8,384**	**2,072**	**42**
**Rm41**	**190**	**7.41**	**20,591**	**7,928**	**1,957**	**39**
T073	266	7.39	12,204	7,827	1,648	87
**T094**	**195**	**7.16**	**21,633**	**7,634**	**1,794**	**31**
USDA1002	359	7.57	8,248	8,268	1,657	202
**USDA1021**	**254**	**7.57**	**14,579**	**8,235**	**1,895**	**56**
HM007-10	216	7.16	20,297	7,576	1,328	28

KH12g	166	6.85	26,202	7,136	1,426	45
KH30a	151	6.90	26,382	7.193	1,358	27
KH35b	150	7.11	33,212	7,418	1,350	22
KH35c	265	7.16	17,701	7.571	1,323	33
KH48e	205	6.87	20,477	7,179	1,431	14
M10	231	6.93	15,018	7,368	1,281	63
T027	187	7.04	19,799	7,356	1,469	102
S. medicae						
**A321**	**221**	**7.26**	**5,277**	**7,684**	**1,633**	**16**
**KH36b**	**178**	**6.93**	**6,276**	**7,459**	**1,437**	**63**
**KH36c**	**179**	**7.24**	**5,716**	**7,787**	**1,492**	**69**
**KH36d**	**182**	**6.94**	**4,880**	**7,461**	**1,428**	**69**
**KH53a**	**175**	**6.89**	**7,712**	**7,444**	**1,389**	**40**
**KH53b**	**164**	**6.89**	**9,486**	**7,460**	**1,408**	**43**
**M1**	**254**	**7.20**	**4,595**	**7,720**	**1,454**	**61**
**M102**	**242**	**7.17**	**4,928**	**7,852**	**1,504**	**63**
**M161**	**221**	**7.26**	**6,019**	**7,815**	**1,517**	**94**
**M2**	**242**	**7.17**	**4,595**	**7,807**	**1,549**	**64**
**M22**	**214**	**7.49**	**5,640**	**8,143**	**1,702**	**14**
**M58**	**162**	**7.02**	**12,660**	**7,647**	**1,590**	**89**
S. saheli						
USDA4893	200	6.18	20,434	6,436	--	--
S. fredii						
USDA205	255	7.18	9,603	7,628	--	--
USDA207	287	6.96	12,280	7,413	--	--
S. terangae						
USDA4894	139	7.10	27,365	7,577	--	--

Table S2. Number of windows duplicated in experimentally-evolved strains descended from three of the strains used in this study. Because the descendants were evolved for < 200 generations in liquid culture, we expect them to have most of the duplications found in their progenitors. The table gives the number of windows duplicated in the progenitor that are also duplicated in 0, 1, 2, 3, 4, or 5 of its descendants. The last column gives the percentage of duplicated windows in the progenitor that were detected as duplicated in all or all but one of the descendant strains.

		Number of duplicated windows that are also duplicated in 0 - 5 descendant strains						
Progenitor	Coverage	0	1	2	3	4	5	Duplicated in all or all but one descendant
USDA1002	1.5	2,476	127	8	13	270		10%
	1.8	1,947	8	10	36	245		13%
	2.0	1,127	37	4	11	245		18%
Rm41	1.5	144	28	32	32	219		55%
	1.8	52	14	19	84	155		74%
	2.0	61	27	20	20	149		61%
KH46c	1.5	64	37	24	19	38	304	70%
	1.8	19	22	17	21	48	258	79%
	2.0	24	2	57	42	66	140	62%

Table S3. Number of 100 bp windows identified as duplicated under 3 levels of minimum coverage (relative to the replicon-wide mean in each strain) in the reference strain as well as the minimum, mean, and median of the 24 *S. meliloti* strains in the focal sub-population. A total of 66,919 windows were analyzed.

Minimum Coverage	Reference strain	min.	mean	median
1.5	208	328	858	579
1.8	10	163	515	375
2.0	6	122	353	273

Table S4. Ratio of the rate (occurrences per bp) of "heterozygous" positions (positions for which the most common base call is supported by < 60% of the reads) in duplicated windows to the rate in unduplicated windows in the 24 *S. meliloti* strains. The "Count" column is the number of duplicated windows (100 bp long) in each strain. Strains marked with an asterisk ("*") are outlier strains.

Cutoff	1.5		1.8		2.0	
Strain	Ratio	Count	Ratio	Count	Ratio	Count
HM006-1*	4.4	2,189	5.4	1,231	8.3	771
HM007-12	3.8	728	5.0	366	3.8	241
HM007-17	1.4	443	1.5	252	0.7	218
HM013-1	25	576	37	290	31	259
HM015-1	42	568	44	490	51	405
KH16b	18	1,180	38	283	34	269
KH46b	46	328	67	209	67	122
KH46c	32	486	37	385	33	332
M156	21	530	16	332	20	166
M162*	24	1,389	36	987	42	391
M195	16	519	28	163	30	145
M210	20	331	21	272	22	223
M243	11	981	18	337	22	211
M249	23	504	22	356	36	139
M268	11	921	25	464	22	299
M270	25	581	29	450	34	365
M30	15	420	23	209	15	188
N6B1	34	708	36	541	31	399
N6B7	24	618	37	383	35	334
Rm41	35	455	46	324	46	277
T073*	9.6	2,154	21	952	29	696
T094	7.7	882	11	385	19	263
USDA1002*	3.6	2,894	4.2	2,246	6.6	1,424
USDA1021	44	495	42	441	33	327
Mean *S. meliloti* (24 strains only)	20.7	858	27.2	515	28.0	353

Table S5. Same as previous table, but for *S. medicae.*

Cutoff	1.5		1.8		2.0	
Strain	Ratio	Count	Ratio	Count	Ratio	Count
A321	55	406	115	189	33	152
KH36b	42	667	42	571	41	481
KH36c	26	890	33	630	34	472
KH36d	24	993	43	627	53	372
KH53a	38	721	45	426	22	268
KH53b	63	596	62	406	68	290
M1	46	1001	55	567	56	403
M102	16	1021	23	587	28	427
M161	30	1124	35	845	47	648
M2	43	1074	53	608	43	520
M22	26	563	63	190	110	98
M58	32	1,639	69	725	74	469
Mean *S. medicae*	36.7	891	53.0	531	50.7	383

Figure S1. Neighbor-joining tree based on concatenated sequences for 645 protein coding genes. The reference strains tare in bold and type strains are in italics. Branch support was assessed using 1000 bootstraps and branches with < 60% support were collapsed to polytomies. For clarity, the bootstrap values are shown only for the deep branches. Scale indicates number of substitutions per site. This figure was redrawn from Sugawara et al. (2013).

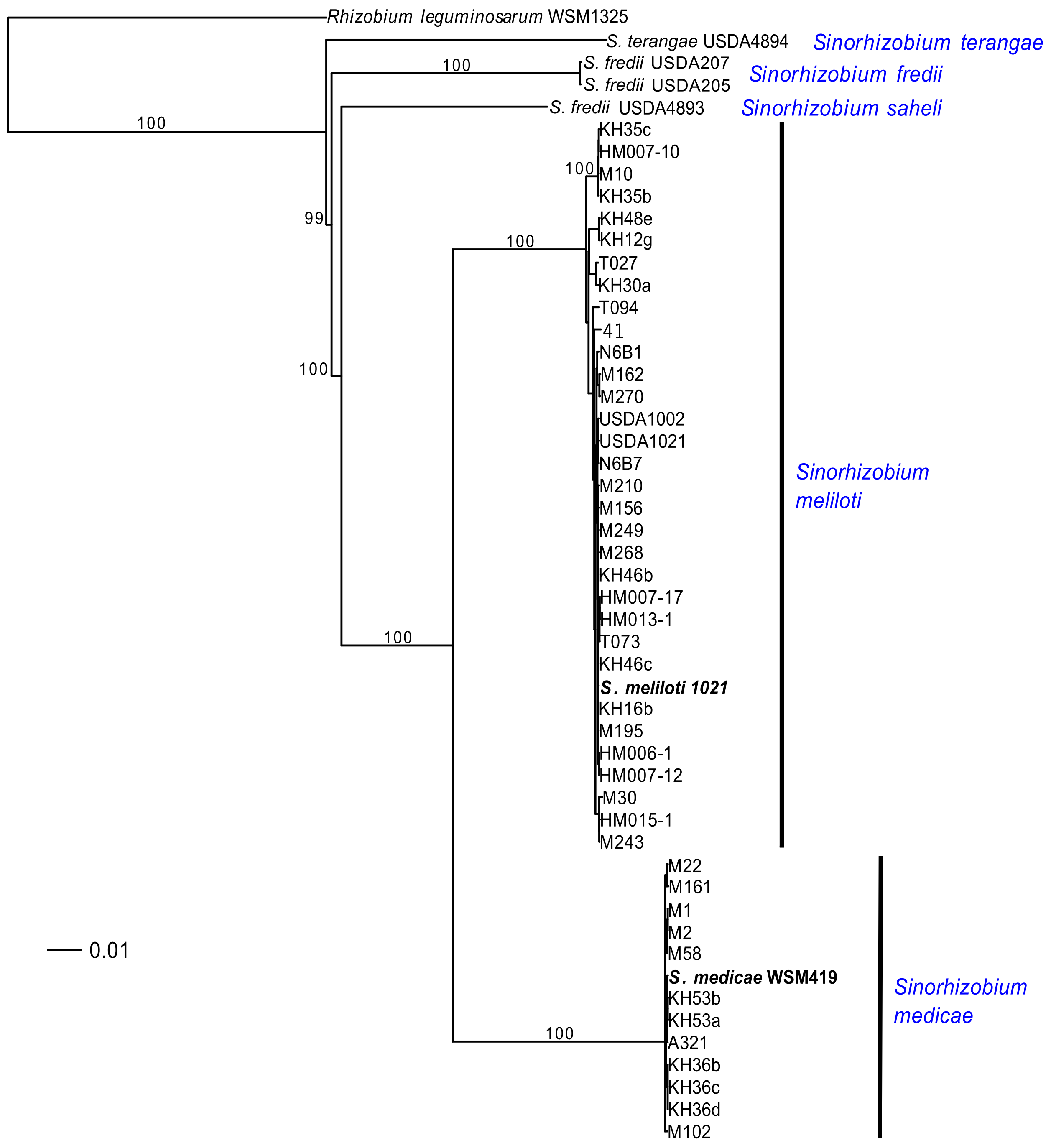

Figure S2. Distribution of Ka/Ks values for duplicated and unduplicated genes. A - C) S. meliloti. D-F) S. medicae. A and D) Chromosome; B and E) pSymA; C and F) pSymB. Ka/Ks values were calculated using the reference genomes.

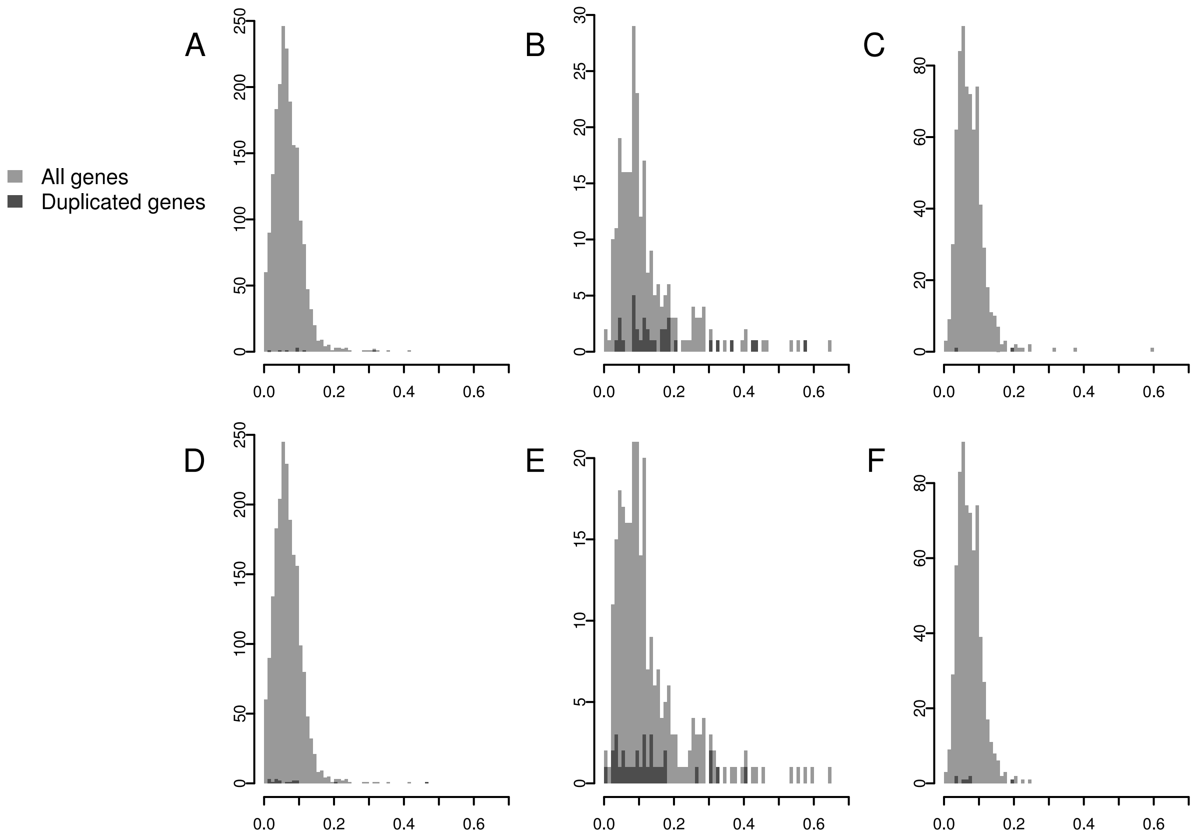

Figure S3. Folded frequency spectra similar to Figure 2, except that only duplications of genes not found in both reference genomes are included. (Differences between types of variants were not tested.)

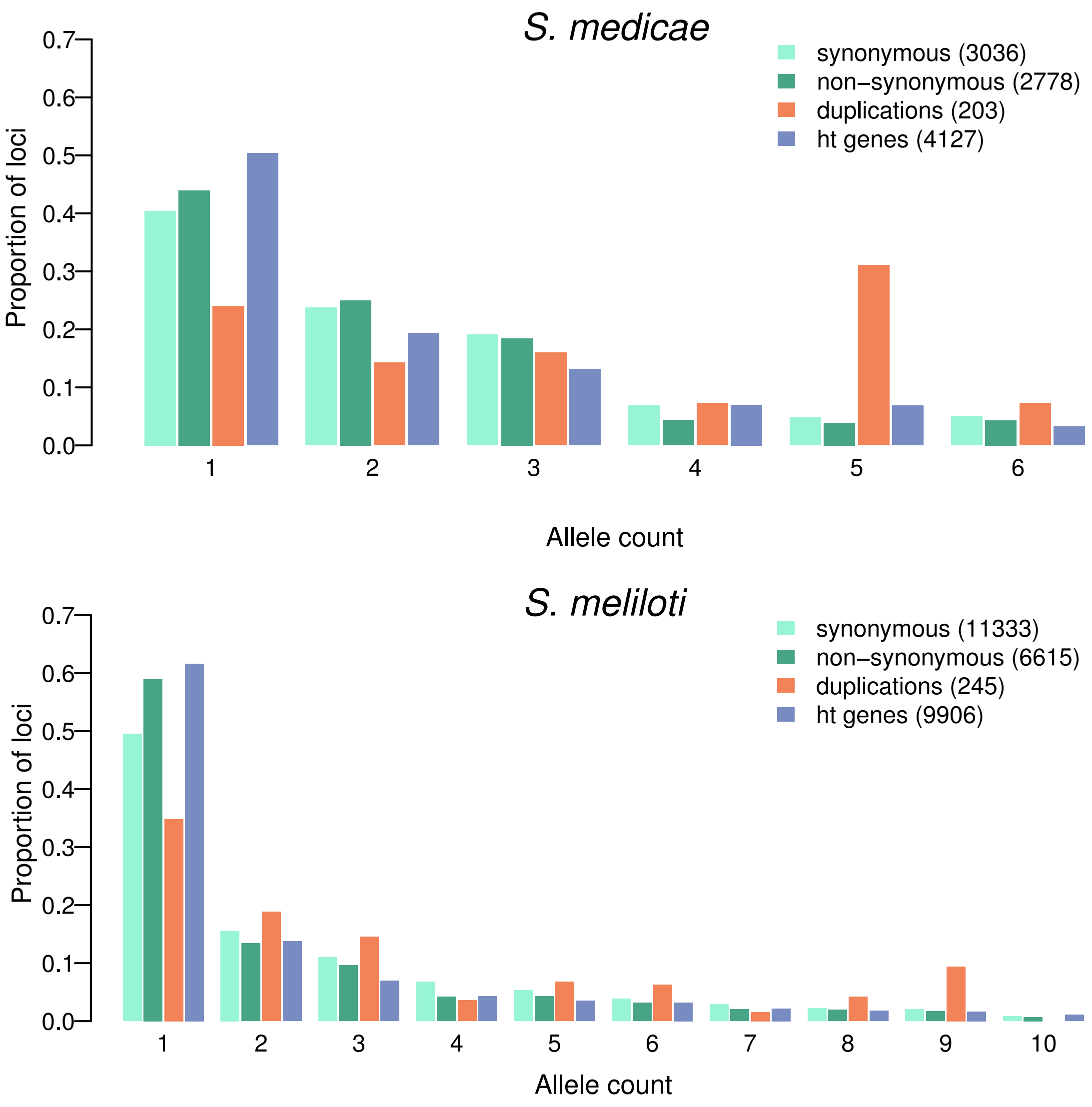

Figure S4. Frequency spectra for derived variants split by replicon.

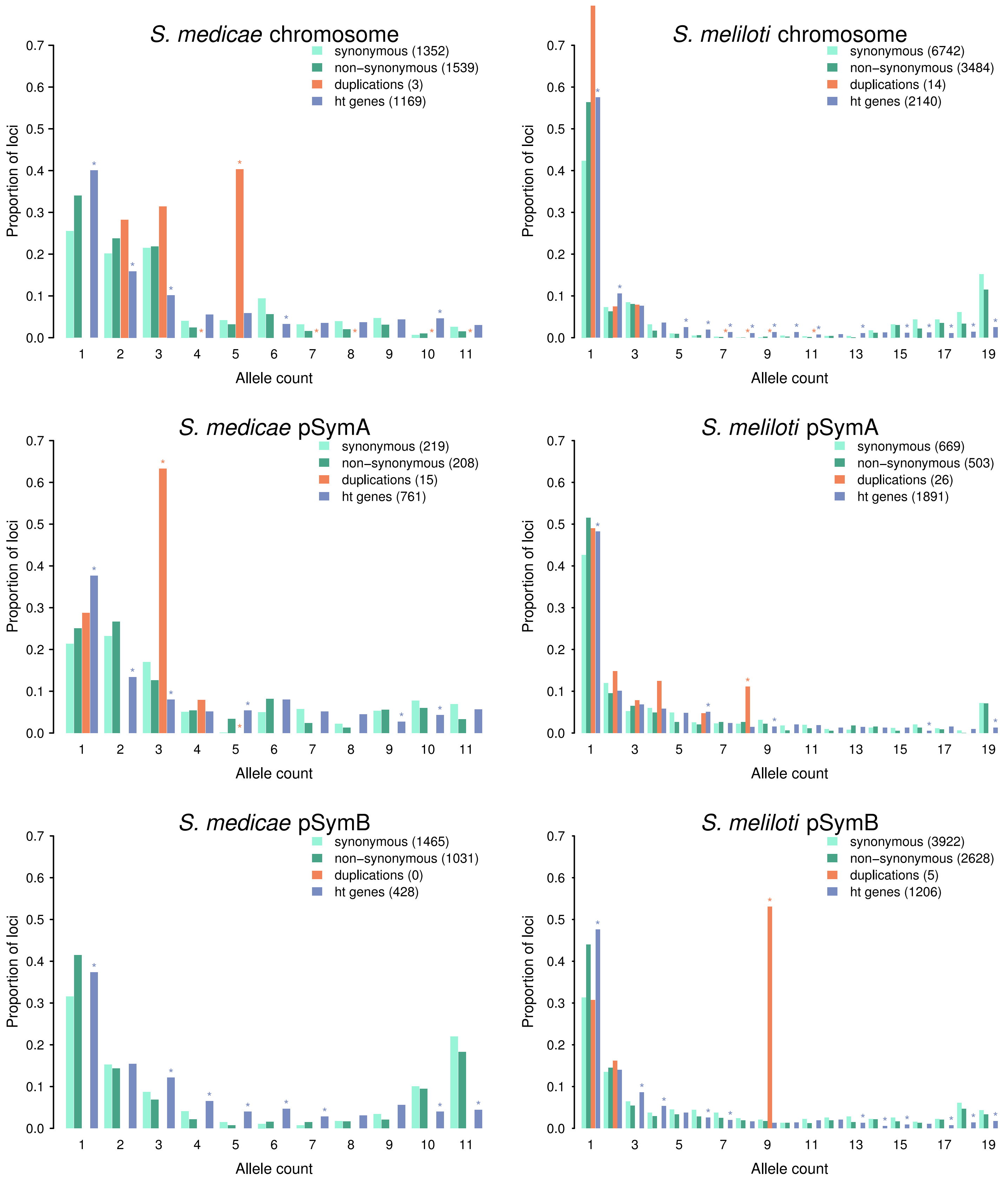

Figure S5. Duplicated 100 bp windows per strain. (A) S. meliloti and (B) S. medicae. Strains are ranked in order of increasing duplications.

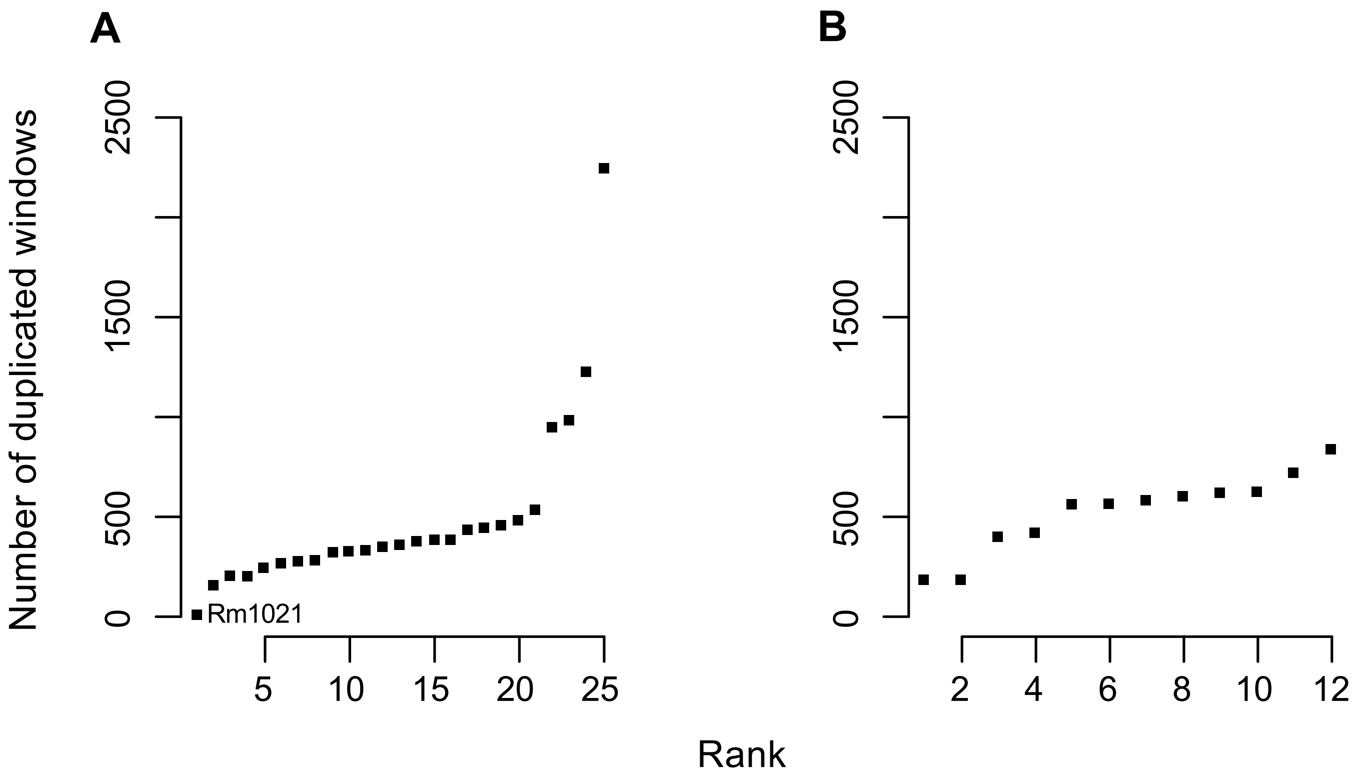

Supplementary References

Bentley DR et al. (194 co-authors). 2008. Accurate whole human genome sequencing using reversible terminator chemistry. Nature. 456:53–59.

Epstein B et al. (10 co-authors). 2012. Population genomics of the facultatively mutualistic bacteria *Sinorhizobium meliloti* and *S. medicae*. PLoS Genet. 8:e1002868.

Epstein B. Population genomics of the legume symbionts *Sinorhizobium meliloti* and *S. medicae*. [PhD thesis]. University of Minnesota. 2013.

Felsenstein J. 1989. PHYLIP -- Phylogeny Inference Package (Version 3.2). Cladistics. 5:164.

Kurtz S, Phillippy A, Delcher AL, Smoot M, Shumway M, Antonescu C, Salzberg SL. 2004. Versatile and open software for comparing large genomes. Genome Biol. 5:R12.

R Development Core Team. 2013. *R: A language and environment for statistical computing*. Vienna, Austria http://www.R-project.org/.

Sugawara M et al. (21 co-authors). 2013. Comparative genomics of the core and accessory genomes of 48 *Sinorhizobium* strains comprising five genospecies. Genome Biol. 14:R17.

Sukumaran J, Holder MT. 2010. DendroPy: a Python library for phylogenetic computing. Bioinformatics 26:1569-1571.

Yoon S, Xuan Z, Makarov V, Ye K, Sebat J. 2009. Sensitive and accurate detection of copy number variants using read depth of coverage. Genome Res. 19:1586–1592.
